# Novel plant breeding techniques to advance nitrogen use efficiency in rice: A review

**DOI:** 10.1080/21645698.2021.1921545

**Published:** 2021-05-25

**Authors:** Sajid Fiaz, Xiukang Wang, Sher Aslam Khan, Sunny Ahmar, Mehmood Ali Noor, Aamir Riaz, Kazim Ali, Farhat Abbas, Freddy Mora-Poblete, Carlos R. Figueroa, Badr Alharthi

**Affiliations:** a Department of Plant Breeding and Genetics, The University of Haripur 22620, Khyber, Pakhtunkhwa, Pakistan; b College of Life Sciences, Yan’an University, Yan’an, Shaanxi, China; c Institute of Biological Sciences, Campus Talca, Universidad deTalca, Talca, Chile; d Institute of Crop Sciences, Chinese Academy of Agricultural Sciences, Key Laboratory of Crop Physiology and Ecology, Ministry of Agriculture, Beijing, China; e State Key Laboratory of Rice Biology, China National Rice Research Institute, Hangzhou, Zhejiang, China; f National Institute for Genomics and Advanced Biotechnology, National Agricultural Research Centre, Islamabad, Pakistan; g Research Center for Ornamental Plants, College of Forestry and Landscape Architecture, South China Agricultural University, Guangzhou, China; h College of Khurma, Taif University, Taif, Saudi Arabia; i College of Science and Engineering, Flinders University, Adelaide, South Australia

**Keywords:** Green revolution, synthetic fertilizers, genome engineering, resource use efficiency, food security

## Abstract

Recently, there has been a remarkable increase in rice production owing to genetic improvement and increase in application of synthetic fertilizers. For sustainable agriculture, there is dire need to maintain a balance between profitability and input cost. To meet the steady growing demands of the farming community, researchers are utilizing all available resources to identify nutrient use efficient germplasm, but with very little success. Therefore, it is essential to understand the underlying genetic mechanism controlling nutrients efficiency, with the nitrogen use efficiency (NUE) being the most important trait. Information regarding genetic factors controlling nitrogen (N) transporters, assimilators, and remobilizers can help to identify candidate germplasms via high-throughput technologies. Large-scale field trials have provided morphological, physiological, and biochemical trait data for the detection of genomic regions controlling NUE. The functional aspects of these attributes are time-consuming, costly, labor-intensive, and less accurate. Therefore, the application of novel plant breeding techniques (NPBTs) with context to genome engineering has opened new avenues of research for crop improvement programs. Most recently, genome editing technologies (GETs) have undergone enormous development with various versions from Cas9, Cpf1, base, and prime editing. These GETs have been vigorously adapted in plant sciences for novel trait development to insure food quantity and quality. Base editing has been successfully applied to improve NUE in rice, demonstrating the potential of GETs to develop germplasms with improved resource use efficiency. NPBTs continue to face regulatory setbacks in some countries due to genome editing being categorized in the same category as genetically modified (GM) crops. Therefore, it is essential to involve all stakeholders in a detailed discussion on NPBTs and to formulate uniform policies tackling biosafety, social, ethical, and environmental concerns. In the current review, we have discussed the genetic mechanism of NUE and NPBTs for crop improvement programs with proof of concepts, transgenic and GET application for the development of NUE germplasms, and regulatory aspects of genome edited crops with future directions considering NUE.

## 1 Introduction

Over the last three decades, global rice production has increased by three fold despite the increases in rice production constraints and input costs. Rice ensures food and nutritional security to more than half of the world’s population.^1^ As such, rice demands a great effort for the development of high-yield, nutritious, climate-resilient, and resource use-efficient varieties to meet the caloric demands of the ever-increasing human population.^2^ Rice production increased remarkably during and after the green revolution period due to the development of high input-responsive rice germplasm. However, the improved germplasm requires more synthetic fertilizers, pesticides, and a frequent supply of irrigation water. Nitrogen (N) is an integral nutrient for plant growth and development.^3^ N is primarily found in photosynthetic metabolites, proteins, and nucleic acids and plays a key role in metabolic and growth-related activities.^4,5^ Cultural and agronomic management practices can help to achieve efficient utilization of N fertilizers. The nitrogen use efficiency (NUE) of several agronomically important crops is of great interest for academia and research. NUE is based on the economic benefit from per unit application of N fertilizer. Several researchers have suggested definitions for NUE; however, it has been unanimously accepted that NUE is the result of N uptake efﬁciency (NUpE) and N utilization efﬁciency (NUtE).^6–8^ Improving NUE enhances crop economics i.e., grain quality, yield, and biomass.^9^ 10 N is primarily applied as synthetic fertilizer while a smaller portion is contributed by grain legumes from N fixation.

**Figure 1.**
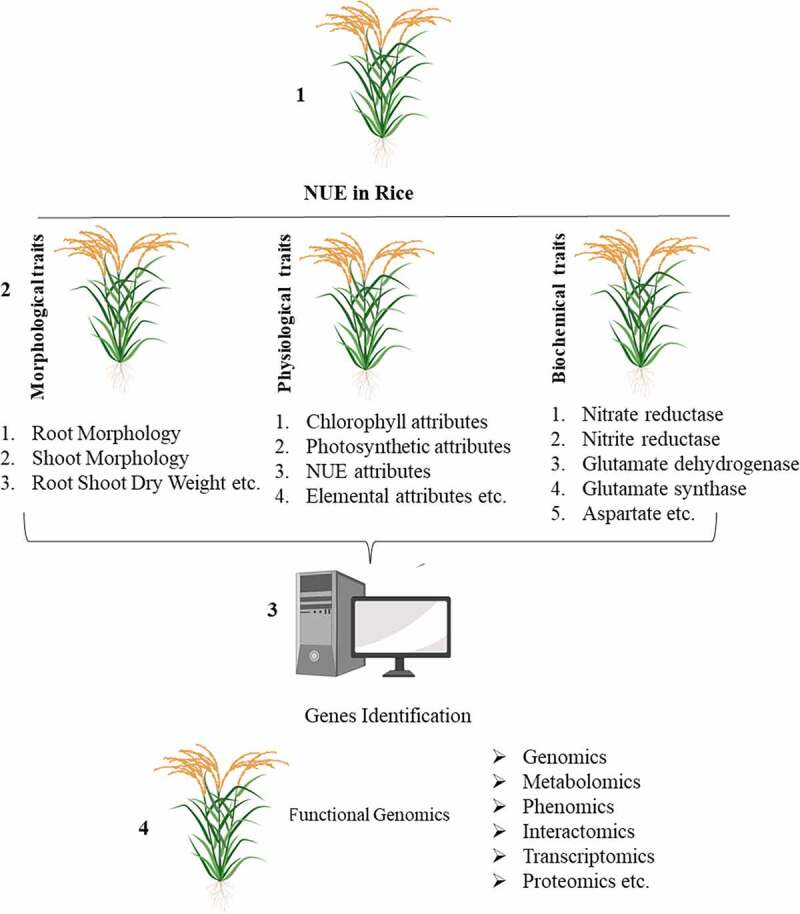
Illustration explaining various traits essential for the identification of NUE genotypes and different OMIC_S_ for the functional characterization of genes controlling NUE in rice.

Plants uptake N through their roots in the form of nitrate (NO_3_^−^) or ammonium (NH_4_^+^) and it is actively utilized to complete metabolic processes.^11^ Being mobile in nature, N losses from soil are greater than any other element; moreover, crop species differ in their N uptake ability. Despite the positive influence of N on yield and yield related components, plants can only uptake 30–50% of the supplied N depending on the soil type, environmental condition, and plant population.^9^ N loss from soil is caused by volatilization, denitrification, and leaching, ultimately polluting the air and water^12–14^ while simultaneously increasing the cost of production.^15^ It has recently been reported that 24–39% of wheat, rice, soybean, and maize production areas have demonstrated stagnation or collapse of yield.^13,16^ Therefore, it is crucial to optimize N fertilizer application or improve crop NUE to achieve high production while also reducing environmental pollution and production cost. Several studies have developed germplasms with improved NUE through classical plant breeding, molecular techniques, and genetic engineering methods.^17^ Moreover, genetic engineering approaches have not yet been extensively adopted compared to traditional breeding techniques for the development of germplasm with improved NUE.^15^ Identifying NUE rice genotypes requires a detailed survey of different morphological, physiological, and biochemical traits, along with functional genetic studies based on various -omics approaches^18^ (Fig. 1).

Genome-editing technology (GET) is a reliable, cost-effective, and versatile approach that has been widely adopted by plant science researchers. The associated efficiency in generating genetic modifications for desirable phenotypes has opened new avenues of research.^19^ However, traditional plant breeding tools and classical GETs have been unable to meet the demands of high precision, efficiency, and timeliness, leading researchers to adapt novel plant breeding techniques (NPBTs). These NPBTs include clustered regularly interspaced short palindromic repeats and CRISPR-associated protein (CRISPR/Cas), CRISPR-CRISPR from Prevotella and Francisella 1 (Cpf1), base editing (BE), and prime editing (PE), and have proven to be powerful tools for successfully modifying genomic sequences in a simple and precise manner.^20^ The use of these GETs gas been reported in several crop plants where desirable phenotypes were successfully obtained. Moreover, NPBTs have enabled the production of transgene-free plants that are categorized as non-genetically modified (GM) crops. The transgene-free plants do not contain exogenous genes and therefore escape the strict regulatory framework of GM crops. The extraordinary NPBTs with reference to GETs are now available and can be utilized for various crop improvement programs to ensure food and nutritional security for the ever-increasing human population. Acknowledging the importance of NUE and recent developments in NPBTs, we provide a non-comprehensive review highlighting the multiple factors influencing NUE and explore how genetic understanding will improve our knowledge of the utility of genetic factors to enhance NUE through various GETs, with a focus on how these GETs can be applied to modify genes to improve NUE in rice. Moreover, detailed information has been provide on the regulatory policies for genome edited crops around the world and future directions with perspective to NUE.

## 2 Genetics Mechanism for NUE

Advances in marker-assisted selection, biotechnological tools, and genomics have helped to reveal that NUE is multigenic in nature. Genomic regions associated with NUE have been investigated in *Arabidopsis*, rice, maize, and wheat.^21–23^ The agronomic attributes, namely grain weight, yield, protein content, and NUE characteristics, namely N harvest index, grain N content, and N remobilization, are considered to be indicators for NUE in plants. In rice, four quantitative trait loci (QTLs) responsible for grain N content have been identified, with two being for shoot N content on chromosomes 8, 9, and 10 under both low and normal N levels. Similarly, two QTLs controlling harvest index and one QTL for physiological NUE on chromosomes 5 and 7 were identified, respectively. In wheat, major QTLs for root NUE, shoot dry weight, and grain yield have been identified.^24^ Moreover, in barley fifteen QTLs influencing NUE have been identified.^25^ The results of studies performed on different crops are challenging to interpret and inconsistent, ultimately requiring larger plant populations, the existence of genetic diversity, and conducting multiple trials across growing seasons.^2^ Moreover, the identified QTLs must be functionally characterized to understand their key roles associated with NUE. The genetic mechanisms of N transporters, assimilators, and remobilizers are discussed in detail below.

### 2.1 Genetic Mechanisms of N Transporters

N in soil is typically available in the form of NO_3_^−^ under aerobic conditions or as NH_4_^+^ in a ﬂooded situation. Mechanistically, these two forms (NO_3_^−ˍ^ and NH_4_^+^) are uptaken from the soil by specialized N transporters involving two physiological phenomena, namely the high-afﬁnity transport system (HATS) and low-afﬁnity transport system (LATS). HATS works under low N concentrations (<250 μM) by employing *Nitrate Transporter 2* (*NRT2*) and *Ammonium Transporter 1* (*AMT1*) for the uptake of NO_3_^−^ and NH_4_^+^, respectively. Whereas, under the LATS system, *NPF* (*NRT1/PTR*) works under elevated N concentrations (>250 μM) to uptake NO_3_^−^ and NH_4_^+^.^26,27^ In rice, ammonium transporters (AMT) grouped into four subgroups from *OsAMT1* to *OsAMT4* among three AMT from *OsAMT1* involved in high-afﬁnity transport, and seven members of *OsAMT2, OsAMT3*, and *OsAMT4* act as low-afﬁnity NH_4_^+^ transporters.^28^ *AMT* transporter proteins are reportedly more effective in improving NUE than nitrate transporters for ammonium-preferring rice cultivars. Given that NO_3_^−^ uptake in rice is much lower than NH_4_^+^, results have suggested that NO_3_^−^ and NH_4_^+^ efficient uptake has the potential to increase NUE along with the improving rice grain production.^15^ In the root zone of rice, ammonium oxidation involves root aerenchyma, thus releasing oxygen which leads to the formation of NO_3_^−^ which is then taken up by plants.^29^ Nitrate transporter1/peptide transporter (*NRT1/PTR*) family genes are recognized as the main transporters for nitrate, amino acids, peptides, glucosinolates, indole-3-acetic acid, abscisic acid, etc.^27^ Genes from this family regulate the transport and allocation of NO_3_^−^ within the plant body organs^30,31^ The *NRT1* and *NRT2* gene families are known as the main regulators of low and high affinity transporters in low nitrate environments.^32^ Over 80 genes have been identified in the *NRT1* and *NRT2* families, but only a select few have were found to belong to the *NRT1* family.^33,34^

### 22 Genetic Mechanisms of N Assimilation

The NH_4_^+^ incorporation into plant cells is reduced to nitrite by the nitrate reductase enzyme in the cytosol.^35^ From there, nitrite is translocated to the plastids and chloroplasts where it is then converted to ammonium by the nitrite reductase (NiR) enzyme. This ammonium derived from nitrate, or that produced by photorespiration or amino acid recycling, is generally assimilated in the plastids by the glutamate synthase (*GS/GOGAT*) cycle.^10^ *GS/GOGAT*, a vital enzyme for N assimilation and remobilization, has two isoforms, namely *GS1*, which is responsible for primary ammonium assimilation in roots or re-assimilation of ammonium in leaves, and *GS2*, which regulates ammonium assimilation in chloroplasts. Among the three *GS* members in rice, *OsGS1.1* and *OsGS1.2* are reportedly expressed in all organs and a reciprocal response to ammonium supply in the rice roots has been observed. *GOGAT* is divided into two types, which differ in their electron donor speciﬁcity, namely *ferredoxin-dependent* (*Fd-GOGAT*) and *NADH*-dependent (*NADH-GOGAT*). Among these, one *ferredoxin* and two *NADH-dependent* enzymes have been identified in rice plants.^36^ To increase the overall NUE of a crop plant, it is essential to increase the N assimilation efficiency. In addition to improvements in N assimilation, carbon (C) assimilation is also a critical factor involving several enzymes; therefore, detecting enzyme activity is essential for the development of rice varieties with high NUE.^37^

### 2.3 Genetic Mechanisms of N Remobilization

**Figure 2.**
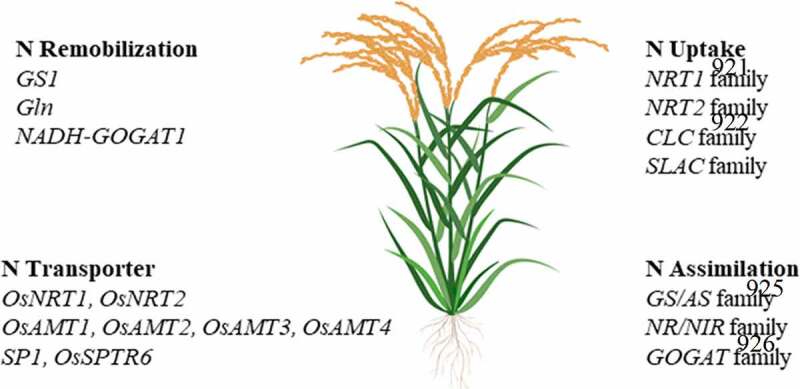
Genes/Gene families involved in plant NUE (modified from.^31,40^

N remobilization is an important process that involves the translocation of N from old senescing plant parts to younger parts during a vegetative stage or into storage organs during the reproductive stage.^38^ Although N remobilization is comprised of various metabolic events, it is a vital process to enhance NUE in crop plants.^10^ In cereal crops, a major contribution of the grain N comes from remobilized N from vegetative organs, and generally this contribution ranges 60–92% depending upon the rate of N remobilization and total N remobilization efficiency. Seed N content is important for proper germination and subsequent germinated seedling growth and establishment. Coordination between N remobilization and senescence-induced protein degradation is required to improve N remobilization efficiency because the onset of senescence triggers the translocation of N to reproductive organs. In rice plants, N translocation from senescing organs accounts for 80% of N in the rice panicle. *GS* and *GOGAT* enzymes are known to regulate this N translocation to reproductive organs.^36^ *GS1.1* and *NADH-GOGAT1* play an important role during the N remobilization process.^36^ Studies have shown that delayed leaf senescence increases grain yields due to an extended photosynthesis duration which contributes more photosynthates to the final grain yields; however, this elongation reduces the rate of N remobilization and subsequently grain N content.^10^ Several genomics studies and QTL analyses have highlighted *GS* involvement in N remobilization efﬁciency in various crops.^39^ Moreover, Fig. 2 shows several genes/gene families that play key roles in controlling components of NUE, such as N remobilization, transporters, uptake, and assimilation, which may prove to be valuable targets for GETs. Moreover, a list of genes available in rice genome has controlling NUE been given in Table 1. Few genes enlisted in Table 1 have been studied through transgenic approaches however, there is need to utilize NPBTs to achieve precision.

**Table 1.** A list of genes available in rice genome controlling NUE

Gene	Function	Source	Reference
*NRT1.1 [NPF 6.3,CHL1)*	Nitrate transporter	*Oryza sativa* L.	^41^
*NRT2.1*	Nitrate transporter	*Oryza sativa* L.	^42^
*NRT3.1 [NAR2.1]*	Nitrate transport component	*Oryza sativa* L.	^43^
*AMT1.1*	Ammonium transporter	*Oryza sativa* L.	^44^
*GS1*	Glutamine synthetase [cytosolic]	*Oryza sativa* L.	^45^
*GS2*	Glutamine synthetase [plastidic]	*Oryza sativa* L.	^46^
*GOGAT*	Glutamate synthase	*Oryza sativa* L.	^47^
*AlaAT*	Aminotransferase	*Oryza sativa* L.	^48^
*ENOD93–1*	Early nodulin	*Oryza sativa* L.	^49^
*OsGOGAT1, OsAMT1*	Increase NUpE in low N conditions	*Oryza sativa* L.	^50^
*glnA*	Grain yield in high, moderate and low N conditions	*Oryza sativa* L.	^51^
*ASN1*	N content in grains	*Oryza sativa* L.	^52^
*SHMT1*	Photosynthesis and grain number per panicle	*Oryza sativa* L.	^53^
*AAP1*	Tiller number and grain yield	*Oryza sativa* L.	^54^
*AAP3*	Tiller number and grain yield	*Oryza sativa* L.	^55^
*AAP5*	Tiller number and grain yield	*Oryza sativa* L.	^56^
*AAP6*	Amino acid uptake from roots, Amino acid transport and grain protein content	*Oryza sativa* L.	^57^
*Pup1/OsPupK46 2/PSTOL1*	Tissue P concentration and relative tiller number	*Oryza sativa* L.	^58^
*qNGR9/DEP1*	Plant height response to N	*Oryza sativa* L.	^59^
*TOND1*	Relative plant dry weight under N-deficient to N-sufficient conditions	*Oryza sativa* L.	^60^
*qNGR2/GRF4*	Ammonium uptake	*Oryza sativa* L.	^61^
*DRO1*	N uptake and leaf N concentration after heading	*Oryza sativa* L.	^62^
*MADS25*	Increase the expressions of NO_3_ ^–^ transporter genes	*Oryza sativa* L.	^63^
*DEP1*	Ammonium uptake and assimilation	*Oryza sativa* L.	^59^
*NAP*	NAC transcription factor positively regulating leaf senescence	*Oryza sativa* L.	^64^

## 3 NPBTs for Targeted Mutagenesis

NPBTs enable plant scientists to make precise modifications to the genome. Classical GETs induce double-strand breaks (DSBs) at a particular genomic location and harnesses non-homologous end joining (NHEJ) and homology-directed repair (HDR) pathways for repair. However, the BE and PE system does not require DSBs for genetic manipulation. These both system holds ability to generated single base pair mutation with more precision. NPBTs are paving the way for further developments to modify the genome for broader objectives, especially for food and nutritional security.

### 3.1 CRISPR/Cas9 Genome Editing System

GETs have revolutionized the field of genetic through efficient and precise manipulation of genomic DNA.^65^ The GETs categorized into first generation i.e., meganucleases, zinc finger nucleases (ZFNs), second generation transcription activator like effector nucleases (TALENs) and third generation includes CRISPR (clustered regulatory interspaced short palindromic repeats)/Cas9 (CRISPR‐associated proteins) and related CRISPR/Cas systems.^66^ In comparison to other GETs, CRISPR/Cas9 system is widely exploited by researchers owing to efficient, accurate, easy in handling and cost effective.^67^ The Cas9 system requires short guide sequence (sgRNA) to direct Cas9 nuclease to cleave the target site.^68^ The Cas9 holds ability to cleave the double stranded DNA target site complementary to sgRNA and successfully deployed various living backgrounds e.g., bacteria,^69^ eukaryotic cells,^70^ animal cells, mammalian system^71,72^ and plants.^73,74^

### 32 CRISPR/Cpf1 Genome Editing System

The CRISPR system from Prevotella and Francisella1 is known as Cpf1, heretofore Cas12a. The CRISPR/Cpf1 gained researchers attention owing to the significant benefits of efficiency and accuracy in genome manipulation.^75^ The Cpf1 endonuclease is comparatively smaller to Cas9 therefore, needs shorter CRISPR RNA (crRNA) with more working efficiency.^76^ Cpf1 binds upstream of the protospacer adjacent motif (PAM) guided by single RNA and cleave the DNA at a distance from the seed region, proximal end of the PAM by introducing staggered cuts of 5 base pair (bp).^77^ The Cpf1 system bypass the need of trans-activating crRNA (tracrRNA) during processing of Cpf1-associated CRISPR repeats to mature into crRNAs.^78^ This mechanism efficiently cut the target region to a short T-rich PAM, however Cas9 system require G-rich PAM sequence. The Cpf1 system keep PAM sequence intact which may vary based on its origin of ortholog whereas, create targeted mutagenesis into the desired DNA. There are several online tools especially Cpf1-database which helps to find the potential target site and design the gRNA in a fast, easy and simple way. Moreover, the online Cpf1-database helps to identify Cpf1 and LbCpf1 through recognition of DNA sequence.^79^

### 33 BE Genome Editing System

Base editing (BE) is a worthy addition to GETs for achieving more efficient genome manipulation with irreversible based conversion at target site. BE is much simpler and precise in nature allowing conversion of nucleotides without formation of DSBs within target DNA.^80,81^ The conversion of cytosine (C) to thymine (T) called cytosine BE (CBE) was firstly developed demonstrated high efficiency.^82,83^ The CBE system consist of four elements i) single sgRNA, ii) dCas9, iii) C deaminase and iv) uracil DNA glycosylase inhibitor (UGI). With the in-depth molecular understanding of deaminases, another system called adenine BE (ABE) developed with conversion efficiency of adenine (A) to guanine (G).^82,84,85^ The BEs restrict indels formation both at target and off-target site/s without requirement of DSBs DNA modification,^86,87^ further allowing single bp conversion i.e., bp substitutions without depending on donor DNA.^80^ Recently, several other BEs have been developed other than CBE and ABE e.g., RBEs (conversion from C to U). In comparison to the previous GETs, BEs proved being more efficient, precise and less time consuming to achieve nucleotide/s substitution in different plant species.

### 3.4 PE Genome Editing System

**Figure 3.**
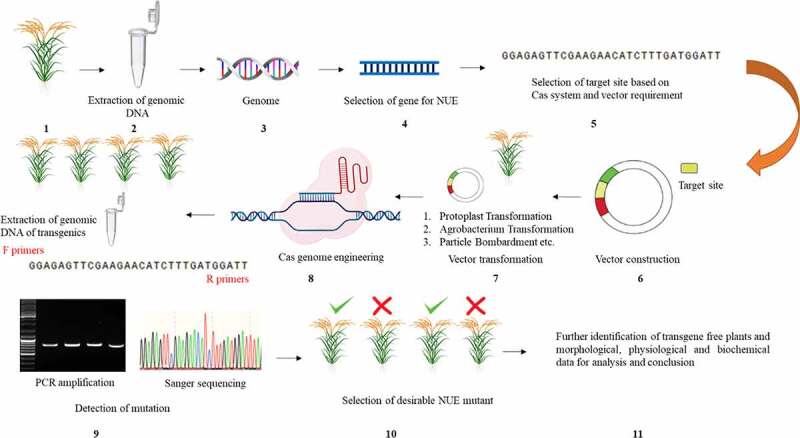
The basic flow chart of genome editing scheme for rice NUE improvement. (1) Selection of desirable germplasm. (2) The extraction of genomic DNA from selected germplasm. (3) Primarily analysis of genome through bioinformatics techniques to identify genes controlling NUE. (4) Selection of gene/genes of interest identified through bioinformatics analysis, available literature/online database. (5) Selection of target site based on GETs and availability/selection of vector. (6) Construction of vector holding gene of interest/target site. (7) Vector transformation through different transformation techniques (protoplast, agrobacterium transformation, and particle bombardment etc. (8) Utilization of Cas genome engineering machinery for targeted modification and extraction of genomic DNA from transgenic plants for mutation identification analysis. (9) The utilization of designed primers for PCR amplification of the target gene site to get Sanger sequencing results. (10) Screening of transgenic mutant plants based on Sanger sequencing results (type of mutation) and phenotypic changes. (11) Selection of transgene-free mutant plants for further collection of (morphological, physiological and biochemical) phenotypic data and interpretation of results.

The recent development in GET has been taken place with the addition of new technique called prime editing (PE). PE technique allows the manipulation of all 12 base-to-base conversion (transition and transversion) bypassing DSBs in targeted DNA.^88^ PE utilize Cas9 nickase bind with reverse transcriptase and PE guide RNA (peg RNA), consist of primer binding site (PBS), target sequence and a sequence to identify the targeted site. The PE system has achieved indels from (approximately 44–80bp), and point mutations with more precision and efficiency. The investigated nine rice and seven wheat lines at protoplasts showed mutation efficiency of approximately 19.2%.^89^ The hybridization of target DNA-pegRNA PBS and target DNA-reverse transcript) resulting minimum off-target effects. The following technique hampered the modification in promoter/introns easier, allowing the allelic replacement in target site feasible. It is noteworthy, the mutation efficiency of PE is similar to BE system however, showed specificity much higher than previously discussed GETs. The PE system is at foundation stage further developments and application for crop improvement program will took place with the passage of time. Lastly, the available literature on GETs application for rice crop improvement has proved it a viable approach to achieve an objective in a shortest period of time. The schematic illustration for the application of GETs for crop improvement program has been described in Fig. 3. The GETs mechanism described and proof of concepts in crop plants established facts to adopt for enhance NUE in rice.

## 4 Transgenic Approaches for Improving NUE

Genetic engineering approaches have been undertaken to enhance NUE in various crops; however, transgenic plants have proven unable to make any significant improvement in NUE for multiple reasons^90,^^7^ Recently, it has been demonstrated that a specific enzyme, GS, is essential for the synthesis of the *Gln* gene, which is responsible for nitrogen recycling and further influences the reduction of nitrogen in pholeum sap in rice.^91^ Over expression of the GS1 enzyme provides a significant improvement in grain yield per plant in rice.^92^ In maize, the knockout of *gln1‐3* and *gln1‐4* resulted in a reduced number of kernels and kernel yield; however, overexpression of *Gln1-3* resulted in a 30% increase in yield.^93^ When *GS1* was over expressed in wheat, a significant improvement in root biomass, along with the number of ears and grains per plant was observed.^94^ Based on these observations in rice, maize, and wheat, it can be assumed that the GS1 enzyme holds significant importance for crop improvement and transgenic approaches can be employed for future studies. The phenotypic expression is largely based on transcription factors involving regulatory networks,^95^ enzymes, transporters, and genes related to NUE that influence nutrient uptake, redistribution, assimilation, and storage.^96^

Previously, *OsAMT1.1* transporter mutant rice were used to increase the NUE in ammonium-preferring rice.^97^ In another study, the ammonium transporter *OsAMT2.1* was expressed under varying nitrogen sources and *OsAMT3.1* was found to exhibit weak expression under the same conditions.^98^ Several studies have investigated ammonium transport in rice via *OsAMT* genes but have had limited success.^99,100^ In rice, *OsNPF8.9* (*OsNRT1*) was characterized as a low-affinity transporter gene responsible for N uptake through root epidermis,^101^ and an increase in N content in rice has been reported in response to its over expression.^102^ Similarly, the *PTR* gene *OsNPF4.1* (*SP1*) is responsible for controlling panicle size^28^; *OsNPF7.3* (*OsPTR6*) is involved in glutamine synthetase and N uptake^30^; *OsNPF6.3* (*OsNRT1.1A*) modulates N utilization within the rice plant^103^ ; and *OsNRT1.1B* regulates nitrate uptake and translocation.^104^ Regarding the high-afﬁnity transporter group, four *NRT2* and two *NAR2* genes have been identified in rice, among which *OsNRT2.3b* and *OsNRT2.4* work independently. In contrast, *OsNRT2.1, OsNRT2.2*, and *OsNRT2.3a* interact with *OsNAR2.1* to regulate nitrate uptake.^105,106^ Moreover, numerous studies attempted to improve NUE through the overexpression of N assimilation genes,^40^ but have had limited success and inconsistent results. Additionally, the transgenic plants must be evaluated under both high and low N conditions. Some studies have demonstrated that over expression of *OsGS1.1* and/or *OsGS1.2* enhances *GS* activities, but there was no signiﬁcant fluctuation in the rice grain yield,^107^ while another study found that over expression of *OsGS1.2* caused an increase in NUE only under the controlled conditions of a growth chamber.^45^ Mutation of *OsNADH-GOGAT2* (like *OsGS1.1*) was found to cause a reduction in spikelet number, growth rate, and grain ﬁlling rate^108^. Additionally, *OsNADH-GOGAT1* (like *OsGS1.2*) mutants have been found to have reduced levels of amino acids and ammonium ions, along with a reduced tiller number.^109^ Over expression of *NADH-GOGAT* has been reported to cause a significant increase in *Indica* rice grain weight,^47^ whereas *OsGS1.3* regulates ammonium assimilation in rice grains.^36^ In rice, three *Gln1* genes were identified that encode *GS1*.^39^ These *Gln1* genes are differentially expressed within the plant body and have different isoforms and functions in different plant tissues.^110^ In this regard, several studies have identified the genes encoding proteins involved in the processes of senescence and N remobilization.^21^ In a study by,^111^ it was found that cytosolic *GS* (*GS1*) re-assimilates ammonium released from protein hydrolysis, which thereby regulates *Gln* synthesis in phloem sap and influences the remobilization efficiency in rice.^91^ Rice mutants lacking *OsGS1.1* exhibited reduced growth and had a decreased rate of grain ﬁlling.^121^ Similarly, *OsGS1.1* has also been found to be involved in mediating glutamine generation, including during the N remobilization process.^112^ *OsGS1.1* is essential for rice growth and yield, while *OsGS1.2* and *OsGS1.3* are unable to compensate for the loss of *OsGS1.1*.^36^

The signaling molecule in plants is NO_3_^−^, but signaling is also influenced by genes such as *AtNPF6.3/NRT1.1* and protein kinases (e.g., *AtCIPK8* and *AtCIPK23*).^113,114^ studied the potential role of *AtNPF6.3/NRT1.1* in N assimilation and plant growth in rice and reported that overexpression of *AtNPF6.3/NRT1.1* elevated the N assimilation under low N concentration. Transcription of *DNA-binding One Zinc Finger* (*DOF*) controls hormone signaling, tissue differentiation, and other biological process in plants.^115^ Transgenic rice for *Zea mays Dof1* (*ZmDof1*) has been developed, and these mutant plants demonstrated increased assimilation of both N and C in the roots, along with an increased photosynthesis rate.^114^ Similar findings were reported by,^17^ where the *FERREDOXIN-NADP+ REDUCTASE* gene was introduced in rice and maize, with the transgenic rice showing increased kernel weight while the transgenic maize displayed improved cob size. The overexpression of the *Dof OsRDD1* gene in transgenic rice enhanced the N responsiveness, resulting high grain yield.^116^ Several studies have documented the key role of the G-protein pathway for N consumption during rice plant development. A major genomic region, *Dense and Erect Panicles 1* (*DEP1*), controls the number of panicles ultimately yielded.^117^ The mutant allele, *dep1*, was found to be associated with the ammonium transporter *OsAMT1.1*, ultimately increasing N uptake.^59,118^ reported a transcription factor, *AtHY5*, responsible for light regulation. Moreover,,^119^evealed the potential role of *AtHY5* in N uptake. In rice, cultivar with high GS activity to recycle NH_3_ leave less NH_3_ compared to cultivars with less GS activity.^120^ Another gene, *DOF18*, induces the ammonium transporters *AMT1, AMT2*, and *AMT3* to influence ammonium uptake from rice root tissue.^53^ Transgenic approaches have been successfully utilized with foreign DNA to develop GM crops that have gone on to pass through strict ethical, social, and biosafety-related regulatory frameworks. Genetic engineering mechanism manipulating genes available in rice genome to improve NUE are listed in Table 2.

**Table 2.** Transgenic approaches manipulating genes controlling amino acid metabolism and transport to improve nitrogen use efficiency in rice

Gene	Source	Promoter used	Phenotype observed	Reference
*PTR6*	*Oryza sativa* L.	*Ubiquitin*	Increased plant growth	^30^
*AMT1.1*	*Oryza sativa* L.	*Ubiquitin*	Increased ammonium, uptake and seed yield	^121^
*AMT2.1*	*Oryza sativa* L.	*CaMV 35S*	Increased Ammonium uptake	^98^
*GS1*	*Oryza sativa* L.	*CaMV 35S*	Increased N, decreased seed yield	^107^
*GS2*	*Oryza sativa* L.	*CaMV 35S*	Photorespiration capacity up	^46^
*GOGAT*	*Oryza sativa* L.	*CaMV 35S*	Increased grain weight	^47^
*AlaAT*	*Hordeum vulgare* L.	*OsAnt1*	Increased biomass and seed yield	^122^
*GDHA*	*Aspergillus*	*CaMV 35S*	Increased DW, N, yield in field	^51^
*DOF1*	*Zea mays* L.	*CaMV 35S*	Increase nitrogen content 30%, enhance growth rate under low N, reduced glucose level	^123^
*ENOD93–1*	*Oryza sativa* L.	*Ubiquitin*	Increased shoot biomass and seed yield	^49^
*glnA*	*Escherichia col*	*CaMV 35S*	Increase grain yield under high, moderate and low N conditions	^124^
*GS1.1, GS2*	*Oryza sativa* L.	*CaMV 35S*	Increase N assimilation and plant biomass	^125^
*OsGOGAT1*	*Oryza sativa* L.	*Activation tagging* *lines*	Increase NUpE in low N conditions; increase N content of grains	^126^
*ASN1*	*Oryza sativa* L.	*Ubiquitin*	Increase N content of grains; no impact on grain yield	^127^
*gdhA*	*Aspergillus niger*	*CaMV 35S*	Increase ammonia assimilation and plant biomass under high N conditions	^51^
*GDH*	*Trichurus*	*Ubiquitin*	Increase N assimilation, thousand grain weight, grain number and seed protein content under high, moderate and low N field conditions	^128^
*SHMT1*	*Oryza sativa* L.	*Actin*	Increased photosynthesis and grain number per panicle	^53^
*ALAAT*	*Hordeum vulgare L.*	*Ant1*	Increase plant biomass, NUpE and final seed yield under high N conditions independently of soil N source [ammonia/nitrate]	^122^
*ALAAT2*	*Cucumis sativa* L.	*Ant1*	Increase NUpE and grain yield in high and moderate N conditions	^129^
*AAP1*	*Oryza sativa* L.	*CaMV 35S*	Increase tiller number and grain yield	^54^
*AAP3*	*Oryza sativa* L.	*CaMV 35S*	Decrease tiller number and grain yield	^55^
*AAP5*	*Oryza sativa* L.	*CaMV 35S*	Decrease tiller number and grain yield	^130^
*AAP6*	*Oryza sativa* L.	*CaMV 35S*	Increase amino acid uptake from roots, amino acid transport and grain protein content at final harvest; maintain grain yield	^57^

## 5 NPBTs for Improving NUE in Rice

The green revolution has proven to be a breakthrough in agricultural production to ensure food and nutritional security. However, the germplasm production potential remains dependent on fertilizer application,^131^ requiring resources to use efficient germplasms.^132^ The recent advances in marker-assisted selection, omics approaches, next-generation sequencing, validation of candidate genes, gene expression analyses, and GETs have aided in the development and screening of potential germplasms to achieve NUE genotypes. Sustainable agriculture requires crop germplasms with premium yield, resistance to biotic and abiotic stresses, environmental resilience, resource use efficiency, and less dependency on artificial fertilizers. The NUE is a ratio of yield to N supply, indicating whether the developed germplasm is lacking in NUE. Conversely, in many parts of the world, especially in developing countries, low-nutrients soils are common and there is often neither the funds nor the infrastructure to provide N-based fertilizers to small farmers. Therefore, geneticists are selecting genotypes/hybrids with the ability for high yield under low N for small farmers. The strong association between yield under high and low N allows breeders to select for broad adaptability in nutrient-replete soils. The classical example of selecting for a plant’s ability to utilize N efﬁciently is Norman Borlaug’s introduction and selection of dwarﬁng genes that resulted in semi-dwarf high yield cultivars. These genes (*Rht-B1* and *Rht-D1*), which were originally derived from a cross between a Japanese variety of dwarf wheat (Norin 10) and a high-yielding American variety (Brevor), became the model for the use of dwarﬁng genes to produce plants that use higher levels of N without the lodging that is common in tall varieties.^133^ The dwarﬁng genes altered stem strength and plant architecture and indirectly generated plants that could produce much higher yield under high (standardized) levels of fertilizer and hence had enhanced NUE.^133^

To resolve the regulatory concerns of transgenics, NPBTs that are faster, more predictable, and can be utilized in a wide range of plant species have been developed.^134^ Genome editing through endonucleases is the most widely adopted technique in plant sciences. The application of various genome editing techniques targeting various traits in different plant species has been described in detail, and enhancing NUE is no exception. A CRISPR/Cas9 APOBEC1 BE system has been used to target one site each from the *NRT1.1B* and *SLR1* genes. The results demonstrated 1.4–11.5% C/T substitution while 1.6–3.9% of the edited plants accounted for C/G replacement. In another study, the BE technology using the rat cytidine deaminase enzyme (APOBEC1) has been successfully employed to induce point mutations in two agriculturally important genes, *NRT1.1B* and *SLR1*, in rice.^135^ *NRT1.1B* encodes a nitrogen transporter and *SLR1* encodes a DELLA protein. As previously reported, a C/T replacement (Thr327Met) in *NRT1.1B* could increase NUE in rice,^104^ and an amino acid substitution in or near its TVHYNP motif results in reduced plant height.^104,136^ The successful application of GETs has demonstrated the potential for improving NUE not only in rice but in many other crops important for food and nutritional security. The availability of genomics data can be further exploited to achieve desirable phenotypic manipulation to support sustainable agricultural development. Based on the successful utilization, it can be assumed that GETs hold the potential for a second green revolution to achieve the United Nations second sustainable development goal of zero hunger. The successful application of genetic engineering approaches and GETs in crop plants are dealt same in several countries and strict regulatory regimes are enforced thus require discussion among all stakeholder involving researchers, policy makers and farming community.

## 6 Regulatory Aspects for Genome Edit Crops

The NPBTs is widely adapted for genome alternation of crop plants. The GETs are a valuable resource for the improvement of agricultural crops to withstand biotic, abiotic stresses and to develop environmentally resilient crops.^20^ The application of NPBTs in plant sciences has raised regulatory concerns both at national and international arena to ensure biological, ecological safety, associated risk management, and legal guidelines on misuse of such sophisticated technologies.^137^ The NPBTs holds potential to resolve global nutritional and food security concern however, there is a need of discussion among various stakeholders to differentiate among transgenic i.e., GM and genome edit crop plants. The anti-GM campaign is based on, i) the insertion of foreign DNA to plant genome causing harmful impact on human health and ii) the insertion of T-DNA with antibiotic resistance genetic factors e.g., Golden Rice and Bt Cotton. These arguments are perfectly resolved through NPBTs, GETs modify the endogenous genes similar to natural variations furthermore, GETs holds ability to introduce the point mutations in any gene of interest, not possible to achieve through classical GETs.^1^ The non-target mutation effects are reduced to minimum level through employing Cas9 variants e.g., Cpf1, base editing and prime editing.^138^ The gene transformation methods in GETs have made these techniques reliable and bio-safe e.g., *Agrobacterium tumefaciens*, a soil-borne bacteria used for gene transformation contain natural DNA, allowing to obtain transgene clean plants to bypass strict GM regulations.^139^

The large debate on GM and Genome edit crops require governmental intervention to formulate clear and uniform regulatory policies. The Cartagena Protocol on Biosafety advanced understanding for the international trade of GM organisms/plants however, still several governments have a divergent opinion on development, commercialization, production and consumption.^140^ Presently, the genome edit crops are dealt with under two regulatory guidelines, i) process-based and, ii) product based.^141,142^ Moreover, the regulation for genome edit crops varies among countries as few nations deal with genome edit crops same as GM others deal with such crops as non-GM.^141^ For instance, United States of America and Brazilian government agreed to regulate genome edit crops similar to developed through conventional breeding,^143^ Canadian regulatory guidelines states any plant based technology to develop new attributes require to go through Canadian Food Inspection Agency regulations.^144^ The Court of Justice of the European Union (ECJ) has declared crops produced via NPBTs regulated the same as GMOs however, traditional mutagenic techniques with established biosafety records are exempted.^145^ To ensure management and risk assessment, the state council of China formulated “Regulation on Administration of Agricultural Genetically Modified Organisms Safety”, categorized genome edit with GM crops.^146^ Similarly, the Indian, Japanese and New Zealand regulatory bodies categorize the genome edit crops similar to GM applying strict biosafety guidelines.^147,148^ Therefore, the already existing regulatory framework in particular countries are applied on genome edit crops. Moreover, the advancements in GETs to produce transgene free plants may help avoid the enforced biosafety related regulations as followed in conventional transgenic plants.^142^ In nutshell, it’s the responsibility of all stakeholders to debate the regulatory framework and came up with uniform regulations promoting the safety of humans, animals, plants and the environment.

## 7 Future Directions

Based on the revolution of molecular biology and the discovery of CRISPR sequences in the microbial immune system, biotechnologists are now able to manipulate the any genome of interest in a specific and precise way. These NPBTs have provided ability to plant scientists for the precise and quick insert/manipulation of desirable traits than conventional breeding.

### 7.1 Gene Regulation

The genome editing techniques has been utilized not only for gene knockout and knockin but also for genetic regulations. The genome regulations primarily consist of activation or repression of genes achieved through fusion of transcriptional activators or repressors with DNA-domains of vector constructs i.e., dCas9, targeting only the regulatory domain of endogenous genes.^73^ Cas9 technology has successfully edited SlCLV3 promoters in *Solanum lycopersicum* generating regulatory mutation.^149^ To modulate the translation of mRNAs the upstream open reading frame of *LsGGP2* resulted tolerant *Lactuca sativa* for oxidative stress and elevated ascorbate content.^81^ The GETs influence the transcript level, and hold ability to manipulate the normal function of non-canonical RNAs for crop improvement. The GETs can engineer transcription mechanism of such RNAs directly to understand their underlying function. Based on these observation, the gene regulation mechanism can be exploited for activation/repression of genomic regions controlling NUE in rice.

### 7.2 Mutant Libraries

The complete genome sequence of several crops e.g., *Oryza sativa, Triticum aestivum, Zea mays, Gossypium hirsutum, Glycine max* is available however, to analyze the functional aspects of genes is challenging in post-genomic era. The 3 K rice genome project has enabled to get genome sequence data of rice mega-varieties grown across large areas and under different ecosystems.^150^ To validate the functional aspects of genes influencing NUE in plant species through GETs are considered as an effective strategy therefore, the high throughput mutant libraries at whole genome level can proved to be a useful resource for elevation of NUE in crop improvement programs.

### 7.3 Multiplexing and Gene Stacking

In plants, the metabolic pathways are responsible for traits with economic importance. These metabolic pathways are controlled by complex genetic networks within a cellular system. Therefore, molecular techniques holding ability to manipulate several genes altogether are of great importance in both basic and applied research.^32^ The GETs allow the genetic manipulation of several genes through multiplexing, editing multiple target sites.^70^ The application of Golden Gate cloning or Gibson Assembly method, multiple gRNAs were assembled driven by different promoters.^151,152^developed a simple strategy to engineer endogenous tRNA through simple and robust method expanding targeting and multiplex editing through CRISPR/Cas9 system. The CRISPR/Cpf1 system had dual nuclease to cleave targeted DNA and its own CRISPR RNA.^153,154^ demonstrated feasibility of multiplex editing in rice through Cpf1 system. Moreover, multiple sgRNAs can also utilized to elevate genome editing in model and non-model crop plants with low gene transformation or induced mutation rate percentage. Therefore, the available genetic information for resource use efficiency in different crop species can be easily manipulated to achieve food security.

### 7.4 Targeted Epigenetic Modification

The advancement in technologies had provided opportunities to investigate chromatin modifications, gene expression, and genome structure.^155^ Plants are heavily dependent on epigenetic modifications to respond to environmental stimuli therefore, these modifications are crucial. The alternation in epigenome can elevate the activities of promoters for genes related to biotic and abiotic stresses moreover, can activate the silent genes to generate novel traits for crop improvement. The epigenetics modification activate the endogenous gene expression through targeting a fusion protein of dCas9 and DNA methyl transferase oracetyl transferase to plant promoters utilizing gRNAs. The methyl transferase function can be altered via dCas9 and gRNA in plant genome target site to modify epigenetic makeup to achieve desirable gene expression. There is few literature describing exploitation of targeted genetic modifications for NUE and it can be recommended for generation of germplasm with improved NUE.

### 7.5 Transgene Free Editing

The introduction of foreign DNA into the plant genome has arisen regulatory concerns and regarded as GMOs.^156^ Following the development of precision genome editing the researchers have focused on the generation of transgene free genome edit plants. The removal of Cas9 gene would also help to reduce the off-target mutation.^157^ Earlier the percentage of transgene clean plants was much lower however, the novel developments e.g., BE and PE systems enabled to generate higher number of transgene clean plants. A combination of BE system and DNA-free editing successfully deployed in wheat with C-to T conversion of 1.8%.^158, 159^ The ability to generate plants without transgene can help to skip strict regulatory regime adapted by several countries.

## 8 Conclusion

The elevation in global rice production at low cost is vital for sustainable food and nutritional security. The improvement in NUE is a key constituent for agronomic, economic and environmental aspects therefore, plant breeders and molecular biologists are taking it as challenge. The NUE being polygenic and complex in nature is a hotspot for dissecting the genetic mechanism through classical and NPBTs in rice. So far, several genomic regions have been identified playing their integral role in controlling NUE. The availability of 3000 rice genome project database can further be utilized to understand the underlying genetic factors influencing the N transportation, assimilation and remobilization. Previously, the transgenic approaches successfully exploited through over expression of genes controlling NUE, thus providing the opportunity to explore negatively regulation genes for the development of resource use efficient crops with better agronomic traits. The recent developments in NPBTs have enabled plant scientists to modify the genome of a model and non-model plant species through targeted engineering of attributes essential for biotic, abiotic stress-resistant, environmentally resilient and resource use efficient crops. The GETs have revolutionized biological research, from novel traits developments, epigenetic modifications, transcription regulation, disease-resistant germplasm, multiplex genome editing and beyond. The continuous effort by researchers enabled utilization technology with more precision, cost-effectiveness and versatility however, the divergent regulatory policies are an obstacle to overcome. There is a dire need to formulate uniform policies addressing biosafety, social and environmental aspects. Based on the proofs of concepts and promising future perspectives it can be concluded that NPBTs hold the potential to be an essential tool for plant breeding programs.
